# Propofol enhances stem-like properties of glioma via GABA_A_R‐dependent Src modulation of ZDHHC5-EZH2 palmitoylation mechanism

**DOI:** 10.1186/s13287-022-03087-5

**Published:** 2022-08-04

**Authors:** Xiaoqing Fan, Meiting Gong, Huihan Yu, Haoran Yang, Sheng Wang, Ruiting Wang

**Affiliations:** 1 Department of Anesthesiology, The First Affiliated Hospital of USTC, Division of Life Sciences and Medicine, University of Science and Technology of China (USTC), No. 17, Lujiang Road, Hefei, 230001 Anhui China; 2 Department of Pathophysiology, School of Basic Medicine, Anhui Medical University, No. 81, Meishan Road, Hefei, 230032 Anhui China; 3 Department of Molecular Pathology, Hefei Cancer Hospital, Chinese Academy of Sciences, No. 350, Shushan Hu Road, Hefei, 230031 Anhui China

**Keywords:** Propofol, Glioma stem cells, GABA_A_R, Src, ZDHHC5, EZH2

## Abstract

**Background:**

Propofol is a commonly used anesthetic. However, its effects on glioma growth and recurrence remain largely unknown.

**Methods:**

The effect of propofol on glioma growth was demonstrated by a series of in vitro and in vivo experiments (spheroidal formation assay, western blotting, and xenograft model). The acyl-biotin exchange method and liquid chromatography-mass spectrometry assays identified palmitoylation proteins mediated by the domain containing the Asp-His-His-Cys family. Western blotting, co-immunoprecipitation, quantitative real-time polymerase chain reaction, co-immunoprecipitation, chromatin immunoprecipitation, and luciferase reporter assays were used to explore the mechanisms of the *γ*-aminobutyric acid receptor (GABA_A_R)/Src/ZDHHC5/EZH2 signaling axis in the effects of propofol on glioma stem cells (GSCs).

**Results:**

We found that treatment with a standard dose of propofol promoted glioma growth in nude mice compared with control or low-dose propofol. Propofol-treated GSCs also led to larger tumor growth in nude mice than did vector-treated tumors. Mechanistically, propofol enhances the stem-like properties of gliomas through GABA_A_R to increase Src expression, thereby enhancing the palmitoylation of ZDHHC5-mediated EZH2 and Oct4 expression.

**Conclusion:**

These results demonstrate that propofol may promote glioma growth through the GABA_A_R-Src-ZDHHC5-EZH2 mechanism and are helpful in guiding the clinical use of propofol to obtain a better patient prognosis after the surgical resection of tumors.

**Supplementary Information:**

The online version contains supplementary material available at 10.1186/s13287-022-03087-5.

## Background

A large proportion of cancers are suitable for surgery, with > 60% of patients undergoing tumor resection. Up to 80% of patients receive anesthesia for diagnosis, treatment, or palliative intervention [1, 2]. Perioperative factors, including anesthesia and surgery, have been reported to be associated with poor outcomes in patients with cancer, including increased rates of mortality and recurrence [3, 4]. General anesthesia is associated with higher rates of tumor recurrence and mortality than regional anesthesia; however, there are conflicting reports [5, 6].

The effect of propofol, a commonly used anesthetic, on tumor prognosis is controversial. In some retrospective studies, propofol, which has anti-inflammatory properties in vitro, was clinically associated with a reduced risk of cancer recurrence compared with sevoflurane anesthesia [7, 8]. Additionally, although some cell culture studies have reported that propofol regulates the proliferation, migration, and invasion of tumor cells, these in vitro studies used propofol over a longer period (e.g., 24–72 h) or multiple doses[9, 10]. Therefore, it is important to establish more clinically relevant models to determine the effects of propofol on tumor growth. Propofol is widely used in glioma resection [11, 12]. However, its effect on glioma recurrence and growth remains unclear.

Propofol is a type A *γ*-aminobutyric acid receptor (GABA_A_R) agonist that exists in the peripheral tissue and has become a tumor-promoting molecule that controls the growth of tumor cells [13, 14]. GABA_A_Rs could enhance growth and promote migration and invasion of papillary thyroid cancer [15]. The gamma-aminobutyric acid (GABA) system exists in peripheral tissues and has been reported to be positively correlated with tumorigenesis. Up-regulation of GABAergic signaling events was revealed in bone marrow lymphocytes in acute lymphoblastic leukemia (ALL) children [16]. Src has been reported to be an important downstream regulator of GABAergic signal transduction [17, 18]. Src is commonly expressed in human cancers, including colon, lung, breast, and endometrial tumors [19, 20]. Notably, Src is a major component of the processes and pathways that regulate glioblastoma (GBM) tumorigenesis, such as proliferation, invasion, migration, and the epidermal growth factor receptor, Ras/Raf/MEK, and PI3K/AKT pathways [2, 21].

Protein S-palmitoylation is a bilateral post-translational modification process that occurs in proteins containing fatty acids and is regulated by protein acyltransferase. It is characterized by a conserved catalytic domain containing Asp-His-His-Cys (DHHC) [22, 23]. Many recent studies have shown that the DHHC protein and its substrates play a key role in tumorigenesis, especially in the development and malignant progression of glioma [24, 25]. ZDHHC5 (Zinc finger DHHC-type containing 5) belongs to palmitoyl acyltransferases (PATs), which has been linked to the development of various cancers. For example, ZDHHC5 stimulates the proliferation and anchorage-dependent and anchorage-independent colony formation of non-small cell lung cancer cell lines and was found to be required in a subset of these cells for establishment of tumor xenografts in mice [26]. Besides, ZDHHC5 is upregulated in p53-mutant glioma cells and promotes their tumorigenicity and invasiveness [25].

In this study, we used an orthotopic tumor model to determine the effect of propofol on glioma growth. We found that propofol activates GABA_A_R to increase Src expression, thereby enhancing ZDHHC5-mediated palmitoylation of EZH2 and Oct4 and promoting the self-renewal and tumor-initiating capacity of glioma stem cells (GSCs).

## Methods

### Ethics statements

This study was approved by the Institutional Review Board of the First Affiliated Hospital of the University of Science and Technology of China (USTC), the Division of Life Sciences and Medicine, USTC. All animal experiments were performed in accordance with the guidelines of the Animal Use and Care Committee of the First Affiliated Hospital of USTC.

### Cell culture and stable cell line generation

U118 and LN18 glioma cell lines were obtained from the Cell Bank of Type Culture Collection of the Chinese Academy of Sciences, Shanghai Institute of Cell Biology, China, and cultured in Dulbecco’s modified Eagle medium (GIBCO) supplemented with 10% fetal bovine serum and 1% (100 ×) penicillin–streptomycin (GIBCO). They were characterized using isozyme detection and DNA fingerprinting.

The U118-derived GSCs and LN18-derived GSCs were derived from U118 and LN18 GBM cells, respectively, and the biological characteristics were analyzed, as shown in Additional file 1: Fig. S1. These GSCs had high stemness marker expression, sphere-formation ability, and tumor-initiating potential. For short-term in vitro amplification of GSCs, the cells were cultured in a Thermo Fisher Scientific medium containing N2 and B27 supplements (Invitrogen), human recombinant basic fibroblast growth factor (Invitrogen, 10 ng/ml), and epidermal growth factor (Invitrogen, 10 ng/ml).

Cells were transfected with Src Stealth siRNA and negative control siRNA (Invitrogen; Carlsbad, CA, USA) at 40 nM using Lipofectamine^®^ RNAiMAX (Invitrogen), according to the manufacturer’s instructions. The sequence of Src Stealth siRNA was chosen as: 5′-CAAGAGCAAGCCCAAGGAUtt‐3′ and 5′-AUCCUUGGGCUUGCUCUUGtt‐3′, and negative control Stealth siRNA was: 5′-UUCUCCGAACGUGUCACGUtt‐3′ and 5′-ACGUGACACGUUCGGAGAAtt‐3′.

A vector with clustered regularly interspaced short palindromic repeats (CRISPR)/Cas9‐mediated knockout (KO) of the GABA_A_R subunit *β*3/*δ* was designed. The sequence of control gRNA was 5-caccgACGGAGGCTAAGCGTCGCAA‐3 and 5‐aaacTTGCGACGCTTAGCCTCCGTc‐3; GABA_A_R sgRNA was 5‐CACCGATAAAAGGCTCGCCTATTCT‐3 and 5‐AAACAGAATAGGCGAGCCTTTTATC‐3′. The gRNA was inserted into the LentiGuid‐EF1a‐Puro vector. Then, the virus was packaged, and U118-derived GSCs were infected with the cas9 overexpression virus. The cells were treated with puromycin (0.5 μg/mL; #A1113802, Thermo Fisher Scientific) to establish stable cell lines for over 1 week. And the results of GABA_A_R*β*3/*δ* KO are shown in Additional file 1: Fig. S2.

### Neurosphere formation

The U118-derived or LN18-derived GSCs were separated into individuals and inoculated with a total of 5000 cells. The 50-µm cell cluster was regarded as a neurosphere, photographed, and trypsinized to cultivate the second generation.

### Immunoprecipitation and western blotting

Cells were collected and lysed in radioimmunoassay buffer supplemented with protease inhibitors, incubated on ice for 30 min, and clarified by centrifugation at 12,000 rpm for 15 min at 4 °C. Total protein lysate (500 μg) was immunoprecipitated with an agarose-fixed antibody at 4 °C. Sodium dodecyl sulfate–polyacrylamide gel electrophoresis (SDS-PAGE) and western blotting were used to analyze the immunoprecipitated and co-immunoprecipitated proteins.

Cells were lysed using a lysis buffer containing a protease inhibitor cocktail (Bio-Rad, CA, USA). Equivalent amounts of cell lysate were dissolved and transferred to a polyvinylidene difluoride membrane (Pall, CA, USA) and incubated with primary antibodies against Sox2 (1:500; R&D Systems), Oct4 (1:500; Abcam), GFAP (1:1,000; Dako), epigenetic modification of histone H3 (H3K27me3) (1:500; Millipore), phospho-Src (Tyr416) (*p*‐Src) (1:1,000; Cell Signaling Technology, Danvers, MA, USA), Src (1:1,000; Cell Signaling Technology), anti-HA-tag (1:1,000; MBL, Japan), Myc‐tag (1:1,000; Cell Signaling Technology), and Flag‐tag (1:1,000; Sigma). The secondary antibodies used were anti-rabbit IgG horseradish peroxidase (HRP)-linked antibody (7074S, 1:3,000; Cell Signaling Technology) and glyceraldehyde 3-phosphate dehydrogenase (GAPDH; 1:1,000; Cell Signaling Technology). The membranes were then incubated with the corresponding HRP-conjugated secondary antibodies (Invitrogen), and the bands were detected using enhanced chemiluminescence (Invitrogen).

### Quantitative real-time polymerase chain reaction (RT-PCR)

Total ribonucleic acid (RNA) was isolated using an RNAqueousTM-Midi Total RNA Isolation Kit (AM1911, Invitrogen), and RT-PCR was performed on a 7300 Cycler (Applied Biosystems) using a VetMAXTM-Plus One-Step RT-PCR kit (4,415,328, Applied Biosystems). Each sample was prepared in triplicate, and the levels of target gene expression were calculated using the 2^−△△Ct^ method, with *β*-actin serving as the internal control. The sequences of gene-specific primers used in the study were ZDHHC4, forward 5′-CCA CTT GGT GAT GTC AG-3′ and reverse 5′-TCC GTG GAA AAG TCA GGA AC-3′; ZDHHC5, forward 5′-ACA CCT CGG CTT GGC TAC TA-3′ and reverse 5′-GTT GGC TCC TTC AAG CTG TC-3′; ZDHHC16, forward 5′-ACG ACT CTG GCA TTT CT-3′ and reverse 5′-GAG ACG GTG GCG ATA TCA TT-3′; ZDHHC17, forward 5′-TGG ATC AAC TTG GAG GGG AC-3′ and reverse 5′-TTT TGC TTG CCT TGC CTC TT-3′; ZDHHC18, forward 5′-CTT CTT CGT CAT GAG CTG CC-3′ and reverse 5′-CTT CTT CGT CAT GAG CTG CC-3′; ZDHHC23, forward 5′-GTC GGG CAG TCT CAA TC-3′ and reverse 5′-TCC TCA CAC AGA TGC CAC AT-3′; cyclin-dependent kinase inhibitor 1 B (CDKN1B), forward 5′-ATG TCA AAC GTG CGA GTG TC-3′ and reverse 5′-TCT CTG CAG TGC TTC TCC AA-3′; runt-related transcription factor 3 (RUNX3), forward 5′-CAG AAG CTG GAG GAC CAG AC-3′ and reverse 5′-TCG GAG AAT GGG TTC AGT TC′; homeobox A5 (HOXA5), forward 5′-GGC TAC AAT GGC ATG GAT CT-3′ and reverse 5′-GCT GGA GTT GCT TAG GGA GTT-3′; and *β*-actin, forward 5′-AGA AAA TCT GGC ACC ACA CC-3′ and reverse 5′-GGG GTG TTG AAG GTC TCA AA-3′.

### Acyl-biotin exchange assay

An acyl-biotin exchange assay was performed to determine the level of protein palmitoylation that occurred. The immunoprecipitated beads were incubated for 1 h at 4 °C in wash buffer (50 mM Tris, pH 7.4, containing 5 mM EDTA, 150 mM NaCl, and 1% Triton X-100) supplemented with 50 mM N-ethylmaleimide. Next, the beads were incubated with 1 M hydroxylamine (pH 7.4) for 1 h at room temperature and exposed to 0.5 μM 1-biotinamido-4-(4-[maleimidoethylcyclohexane]-carboxamido) butane (pH 6.2) for 1 h at 4 °C. The samples were analyzed by SDS-PAGE and immunoblotting.

### DNA preparation and bisulfite genomic sequencing

Genomic DNA was extracted using the PureLink™ Genomic DNA Mini Kit (Invitrogen), and sodium bisulfite treatment of the extracted DNA was performed as previously described [27, 28]. Primers (forward 5-GGA TTT GTA TTG AGG TTT TGG AG-3, reverse 5-TAA CCC ATC ACC TCC ACC AC-3) were designed to amplify the Oct4 promoter region and exon 1 from − 234 to + 46 for bisulfite genomic sequencing. The amplified products were purified using the PureLink Pro 96 Genomic DNA Purification Kit (Invitrogen), subjected to TA cloning using the pEASY-T3 vector (TransGen Biotech), and sequenced. Cytosine or thymine residues at CpG sites were methylated and unmethylated, respectively.

### Chromatin immunoprecipitation (ChIP)

ChIP assays were performed according to the manufacturer’s instructions (17-10086; Merck Millipore, Sigma-Aldrich, St. Louis, MO, USA). Cells (1 × 10^7^) were treated with 1% formaldehyde for 10 min to cross-link histones to DNA. After sonication of the cell pellets, the lysate was incubated with 10 μL of anti-Src or anti-p53 antibody. To collect immunoprecipitated complexes, magnetic beads were added and incubated with the lysate overnight at 4 °C. After the cross-linking was reversed, the DNA was extracted and purified using the phenol/chloroform method, ethanol-precipitated, and dissolved in water. The ChIP products were assayed via SYBR Green ChIP-qPCR using the following set of primers: (forward) GCA GAA ATA CCT CAC CAA GTT TTT A and (reverse) TTT GGC ATA CTT ACA GAC ACA AGA C.

### Src ubiquitylation assay

To analyze the effects of GABA_A_R and propofol on the regulation of Src ubiquitylation, Src‐Flag and ubiquitin‐HA vectors were transfected into U118-derived GSCs using Lipofectamine 3000 (Invitrogen). Then, 24 h later, cells were treated with propofol for 3 h. Protein lysates were obtained from the cells lysed with lysis buffer (Pierce IP lysis buffer, Thermo Fisher Scientific) and incubated with anti-Flag gel beads (EZview Red Anti‐Flag Affinity Gel, Sigma) at 4 °C overnight. The beads were then washed three times with lysis buffer. The proteins were released from the beads for western blotting and were analyzed using an anti-HA antibody (MBL) to evaluate ubiquitylation.

### Cycloheximide chase assay

U118-derived GSCs were treated with propofol or a control condition for 3 h. Then, the medium was replaced, and cycloheximide (CHX; final concentration 50 µg/ml) (Selleck) was added for the indicated times (0, 4, 8, or 12 h). Finally, cells were lysed for western blotting to detect the Src protein.

### Animal experiments

GBM0378 was isolated from surgical specimens in an earlier study and confirmed [29]. U118-derived GSCs overexpressing luciferase were treated with propofol (Sigma-Aldrich, St. Louis, MO) (4 µg/ml, the clinically relevant blood concentration of propofol) or DMSO (vehicle control) (Sigma-Aldrich, St. Louis, MO) for 6 h in culture dishes. 2 million of GBM0378 cells (2 μL, containing luciferase) or 1000 U118-derived GSCs in phosphate-buffered saline with high glucose were stereotactically injected to the hemi-striatum of six-week-old female BALB/c mice (18–25 g) pre-anesthetized by the intraperitoneal administration of ketamine (132 mg/kg) and xylazine (8.8 mg/kg). The coordinate parameters adopted were as follows: dorsoventral =  – 3.5 mm; medio-lateral =  + 2.5 mm; and antero-posterior = 0.

The dose of 20 or 240 mg/kg of propofol was chosen according to the methods described in previous studies [10, 30], with modifications. Specifically, for mice in the low‐dose propofol group, 20 mg/kg (about 0.5 mg propofol in 50 µL intralipid per mouse) propofol was injected. The same procedure was repeated for mice in the standard-dose propofol group; however, 3 min after the administration of 20 mg/kg propofol, an angel catheter (size 26G, Zibo Eastmed Healthcare Products Co., Ltd., Zibo, China) was placed in the mouse tail vein. A syringe pump (WH‐SP‐08, Wenhao Microfluidic Technology Co., Ltd., Suzhou, China) was used to inject propofol (240 mg/kg) slowly (over 1 h).

Luciferin was injected into the peritoneal cavity to track tumor cells in vivo over a post-injection period of approximately 6 weeks. Animals were anesthetized with sodium pentobarbital (50 mg/kg), and the IVIS Lumina system was used for bioluminescence imaging (PerkinElmer).

### Statistical analysis

All grouped data are presented as the mean ± standard error. Between-group comparisons were analyzed using Student’s *t* test or one-way analysis of variance using GraphPad Prism version 8 (GraphPad Software, La Jolla, CA, USA). All experiments were repeated for each specimen with at least three biological replicates. The criterion for significance (*p *values) was set as described in the figures.

## Results

### Propofol increased the self-renewal and tumor-initiating properties of GSCs

**Fig. 1:**
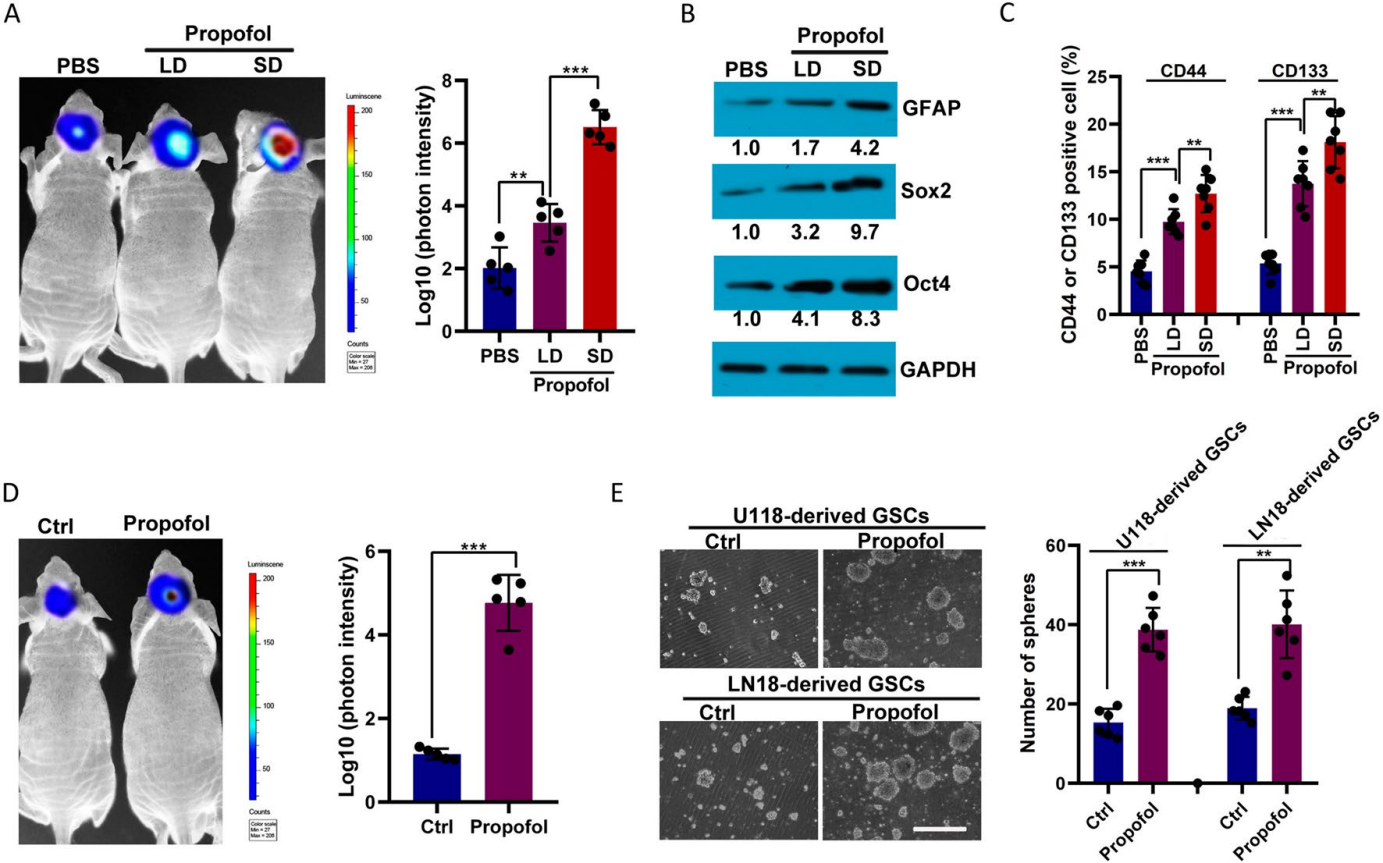
Propofol increases glioma growth by enhancing the stem-like properties of tumors. **A** GBM patient-derived xenografts (PDX; GBM0378) mouse model injected with low and standard doses of propofol by ex vivo bioluminescent assay. Quantification of bioluminescent photon intensity (*n* = 5 in each group). **B** Detection of GFAP, Sox2, and Oct4 in the tumor of a PDX mouse model injected with or without low or standard doses of propofol by western blot. **C** Detection of CD44 and CD133 in the tumor of a PDX mouse model injected with or without low or standard doses of propofol by flow cytometry. **D** Ex vivo bioluminescent assay comparing glioma growth in mice injected with propofol-treated or DMSO-treated glioma stem cells (GSCs). Quantification of bioluminescent photon intensity (*n* = 5 in each group). **E** Detection of glioma spheres formed for U118-dervied and LN18-dervied GSCs treated with or without propofol and cultured for a second passage. Scale bars, 100 μm. Quantification of glioma spheres formation capacity (*n* = 5 in each group). Ctrl, control; LD, low dose; and SD, standard dose

We orthotopically injected patient-derived (PDX) tumor xenolines (GBM0378) into the hippocampus of nude mice and, subsequently, a low or standard dose of propofol into mice via the tail vein. Ex vivo bioluminescence imaging of the mouse brain 4 weeks after the injections showed that treatment with propofol led to significant increases in tumor growth, particularly at a standard dose (240 mg/kg/h, over 1 h), compared to no treatment or treatment with low-dose propofol (20 mg/kg) (Fig. 1A). Propofol treatment upregulated the levels of the glioma marker, GFAP, and glioma stem cell markers, Oct4 and Sox2, expressed in PDX tumors (Fig. 1B). Consistent with these results, flow cytometry analysis showed that the GSC markers CD133 and CD44 were significantly upregulated in PDX tumors after propofol treatment (Fig. 1C). These data suggest that propofol may promote tumor cell growth in vivo, especially in GSCs.

Next, we compared the effects of propofol anesthesia versus non-anesthesia on GSC tumor-initiating potential by injecting propofol-treated (6 h) versus vehicle (DMSO)-treated GSCs derived from U118 cells (U118-derived GSCs) into nude mice. We found that propofol-treated GSC had increased tumor growth compared with DMSO-treated tumor cells (vehicle control condition) 4 weeks after injection (Fig. 1D). These results suggest that short in vitro propofol treatment was sufficient to increase the number of tumor-initiating cells in all GSC samples, resulting in accelerated tumor development.

To further study the role of propofol in GSC biology, we derived GSC cultures from U118 and LN18 GBM cell lines (U118-dervied GSCs and LN18-derived GSCs, respectively) by allowing expansion under serum-free conditions, under which single-cell suspensions were freshly isolated into cellular spheroids. Under free-floating neurosphere culture conditions, propofol treatment increased the capacity of GSCs to form neurospheres (Fig. 1E). Consistent with this, the proliferation, metabolic activity, and stemness gene expression of GSCs were enhanced after propofol treatment (Additional file 1: Fig. S3). These results further indicate the promoting role of propofol in GSC self-renewal and tumor-initiating capacity.

### Propofol increased EZH2 palmitoylation

**Fig. 2:**
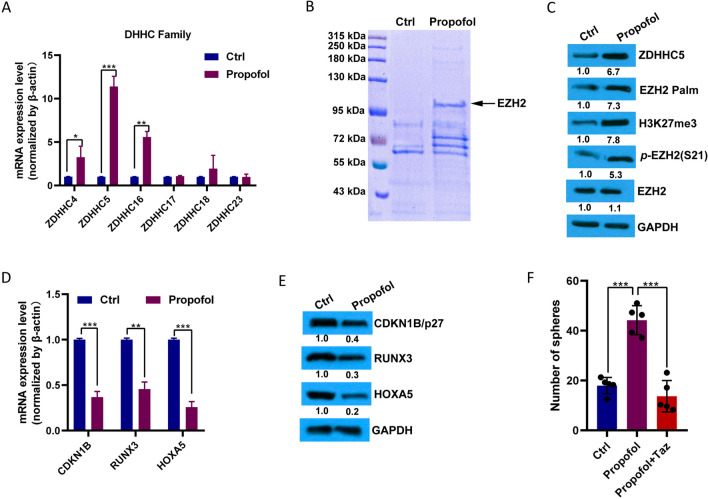
Propofol increases EZH2 palmitoylation mediated by ZDHHC5. **A** RT-PCR analysis of the mRNA levels of 24 known DHHCs in U118-derived GSCs treated with or without propofol (*n* = 5 in each group). **B** Lysates of propofol-treated U118-derived GSCs were subjected to the acyl-biotinyl exchange method (ABE) with a streptavidin biotinylated antibody. Palmitoylated proteins were subjected to LC–MS/MS analysis. The identified peptide sequences of EZH2 are shown. **C** Acyl-biotinyl exchange method and western blotting for palmitoylated EZH2 and western blotting for ZDHHC5, *p*-EZH2 S21, and H3K27me3 in U118-derived GSCs treated with or without propofol (*n* = 5 in each group). GAPDH was used as a loading control. **D** The mRNA levels of CDKN1B, RUNX3, and HOXA5 in U118-derived GSCs treated with or without propofol were analyzed by RT-PCR (*n* = 5 in each group). *β*-actin was used as the loading control. **E** Western blotting for CDKN1B, RUNX3, and HOXA5in U118-derived GSCs treated with or without propofol (*n* = 5 in each group). GAPDH was used as a loading control. **F** Detection of glioma spheres formed for U118-derived GSCs treated with or without propofol, and EZH2 inhibitor, tazemetostat, and cultured for a second passage. Scale bars, 100 μm. Quantification of glioma spheres formation capacity (*n* = 5 in each group). Ctrl, control; Taz, tazemetostat

Several DHHC family members play a key role in glioma tumorigenesis [29, 31]. We analyzed DHHC expression in GSCs, with or without propofol treatment, using RT-PCR. Of the 24 protein acyltransferases, six were related to gliomagenesis [32]. ZDHHC5 was significantly upregulated in propofol-treated GSCs compared to control GSCs (Fig. 2A). We also used an acyl-biotinyl exchange method with a streptavidin biotinylated antibody to purify palmitoylated proteins in propofol-treated GSCs, and the components were analyzed by liquid chromatograph-mass spectrometer-mass spectrometry (LC–MS/MS). Peptide sequences of EZH2 were identified, and the abundance of EZH2 was higher in propofol-treated GSCs than in control GSCs (Fig. 2B, and Additional file 2: Table S1). Furthermore, an increase in ZDHHC5 augmented EZH2 palmitoylation, which, in turn, altered the phosphorylation status of EZH2 S21 (Fig. 2C). By catalyzing H3K27me3, EZH2 represses a series of tumor suppressor genes associated with the tumorigenic properties of glioblastoma, including CDKN1B, RUNX3, and HOXA5 [33]. Therefore, we assessed the EZH2-mediated gene expression. The mRNA levels of these genes were reduced (Fig. 2D and E). Moreover, injection of the EZH2 inhibitor tazemetostat into GSCs treated with propofol markedly reduced tumor size (Fig. 2F). These results indicate that EZH2 palmitoylation, mediated by ZDHHC5, contributes to the development of GSCs after propofol treatment.

### Propofol increasedOct4 expression through the weakening of Dnmt3A and Oct4 promoter binding

**Fig. 3:**
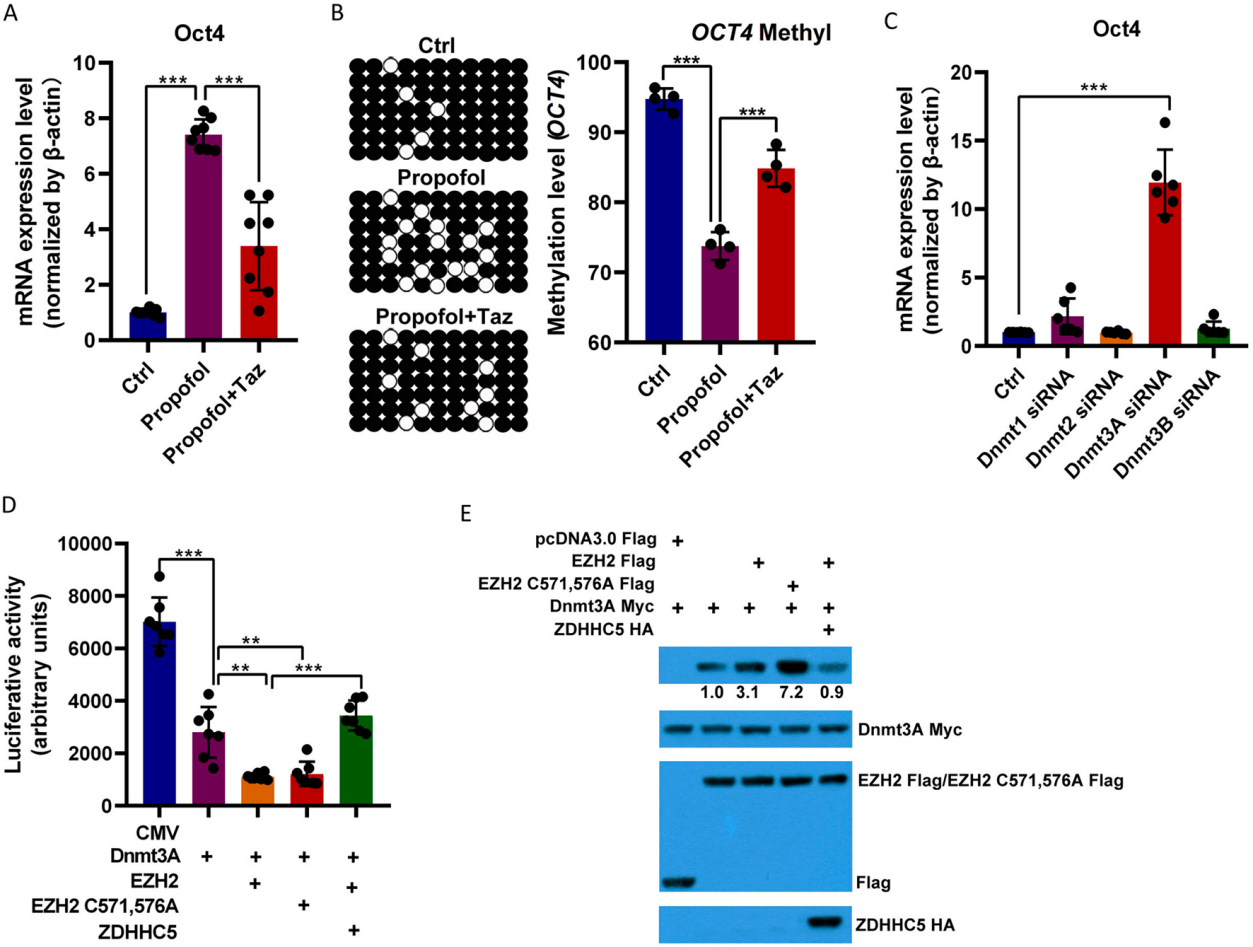
Propofol increases Oct4 expression via the downregulation of the methylation levels of its promoter. **A** The mRNA levels of Oct4 in U118-derived GSCs treated with or without propofol and EZH2 inhibitor, tazemetostat were analyzed by RT-PCR (*n* = 5 in each group). *β*-actin was used as the loading control. **B** The DNA methylation levels of the Oct4 promoter in U118-derived GSCs treated with or without propofol were determined by bisulfite genomic sequencing (*n* = 7). Black and white circles represent methylated and unmethylated sites, respectively. **C** The mRNA levels of Oct4 in U118-derived GSCs transfected with control, Dnmt1, Dnmt2, Dnmt3A, or Dnmt3B siRNA (*n* = 5 in each group). *β*-actin was used as the loading control. **D** Luciferase reporter assays with the wild-type Oct4 reporter vector and DNMT3A, EZH2 (or EZH2 palmitoylation mutants), and ZDHHC5, alone or in combination, in U118-derived GSCs. The luciferase values were relative to Renilla luciferase activity (*n* = 7 in each group). **E** Lysates from 293 T cells expressing Flag-EZH2 palmitoylation mutants (EZH2 C571, 576A), HA-ZDHCH5, and Myc-DNMT3A were subjected to immunoprecipitation, followed by immunoblotting with anti-Flag, anti-Myc, and anti-HA antibodies. Ctrl, control; Taz, tazemetostat

The protein (Fig. 1B) and mRNA (Fig. 3A) levels of Oct4 were also upregulated in propofol-treated GSCs, possibly because of the downregulated methylation of the Oct4 promoter (Fig. 3B). DNA methylation levels in propofol-treated GSCs (94.82 ± 16.38%) decreased noticeably compared with that in untreated GSC (74.03 ± 19.21%). Oct4 expression was suppressed, and its methylation level was upregulated (88.31 ± 9.26%) when EZH2 was knockdown in propofol-treated GSCs. Next, we analyzed the effect of the DNA methyltransferase (DNMT) family on Oct4 expression and found that only DNMT3A knockdown increased the expression of Oct4, whereas other DNMT family members did not (Fig. 3C). EZH2 promoted the binding of DNMT3A to the Oct4 promoter and then methylated the Oct4 promoter to inhibit its expression (Fig. 3D and E). This process may be inhibited by EZH2 palmitoylation, and if EZH2 palmitoylation sites were mutated, their binding would be further enhanced. Thus, these results indicate that propofol increased EZH2 palmitoylation mediated by ZDHHC5, that EZH2 palmitoylation may suppress the binding of DNMT3A and Oct4, that the methylation of the Oct4 promoter region was weakened, and that the expression of Oct4 was enhanced.

### Propofol increased ZDHHC5 by enhancing Src and p53 interaction

**Fig. 4:**
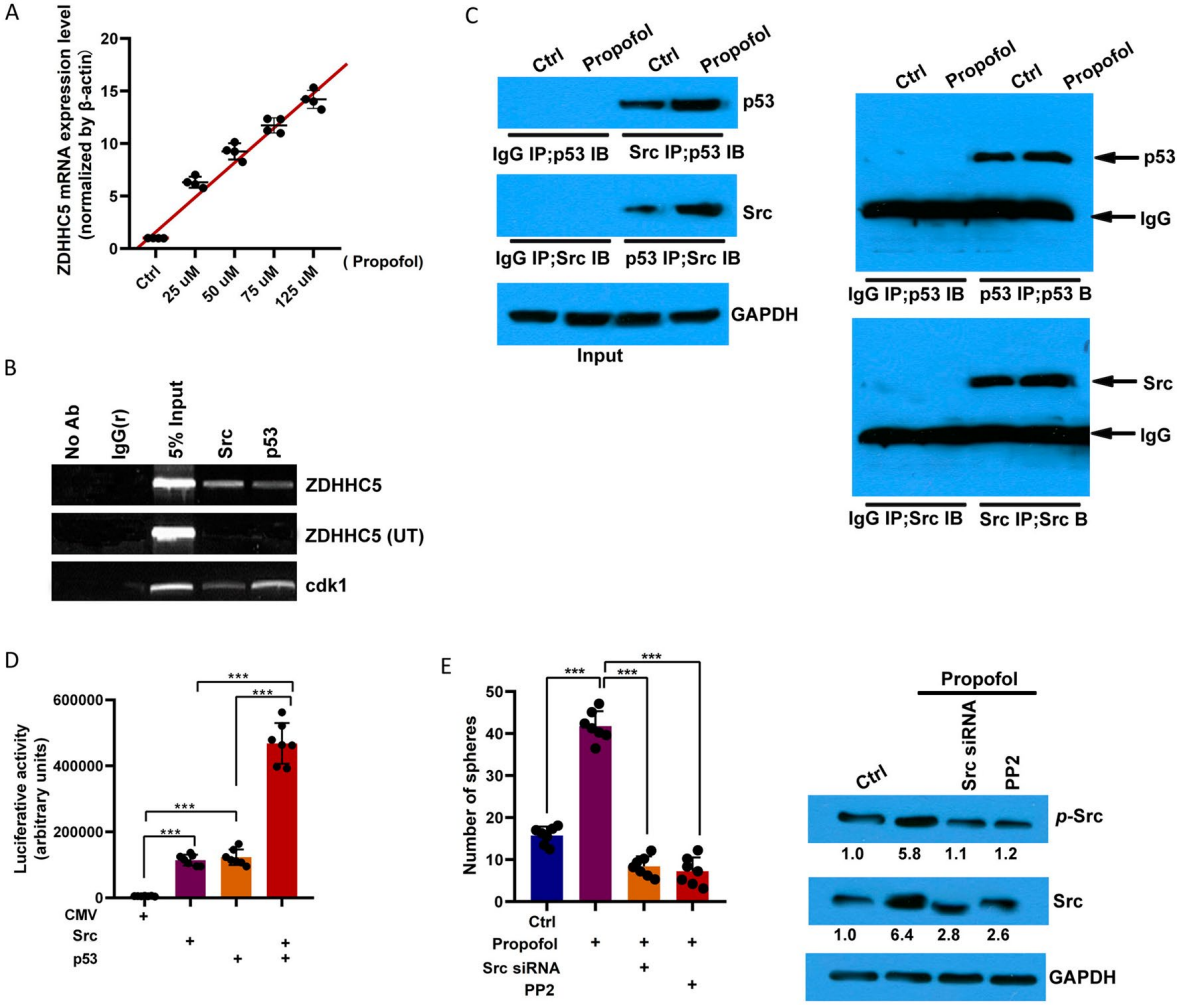
Propofol upregulates ZDHHC5 transcripts by increasing the Src and p53 interaction. **A** The mRNA levels of ZDHHC5 in U118-derived GSCs treated with propofol in different concentrations were analyzed by RT-PCR (*n* = 5 in each group). *β*-actin was used as the loading control. **B** ChIP was performed on U118-derived GSCs with the indicated p53 and Src antibodies. PCR analysis was performed on the endogenous promoters of ZDHHC5 and cdk1 genes; cdk1 was used as a positive control; and the primers for unrelated target sites of ZDHHC5 (ZDHHC5 NT) were used as a negative control. **C** An immunoprecipitation assay was performed on U118-derived GSCs with the indicated p53 and Src antibodies. **D** Luciferase reporter assays with the wild-type ZDHHC5 reporter vector, p53 and Src, alone or in combination, in U118-derived GSCs. The luciferase values were relative to the Renilla luciferase activity (*n* = 7 in each group). **E** Detection of glioma spheres formed for U118-derived GSCs treated with or without propofol, Src siRNA or Src inhibitor, PP2, and cultured for a second passage. Quantification of the glioma sphere-formation capacity (*n* = 5 in each group). Western blotting for *p*-Src and Src in GSCs treated with or without propofol, Src siRNA or Src inhibitor, PP2 (*n* = 5 in each group). GAPDH was used as a loading control

RT-PCR analyses revealed elevated levels of ZDHHC5 transcripts in propofol-treated GSCs in a concentration-dependent manner (Fig. 4A). Of note, through bioinformatics analysis using JASPAR, we discovered that the 1-kb upstream region of the ZDHHC5 promoter contained four potential Src/p53 binding sites (Additional file 1: Fig. S4), and ChIP revealed a direct association between p53 or Src protein and these regulatory sequences (Fig. 4B). After propofol treatment, the expression of Src was upregulated, and the interaction between Src and p53 was enhanced (Fig. 4C). A luciferase assay also indicated that the ZDHHC5 promoter was promoted by Src or p53 overexpression, and co-overexpression of Src and p53 further increased ZDHHC5 transcription (Fig. 4D). Consistent with these results, the capacity of GSCs to form neurospheres was diminished by a Src inhibitor (PP2) or siRNA, even after propofol treatment (Fig. 4E). These results demonstrate that propofol promoted the expression of Src and its combination with p53 and regulated ZDHHC5 expression.

### Propofol suppressed GABAAR-dependent Src expression

**Fig. 5:**
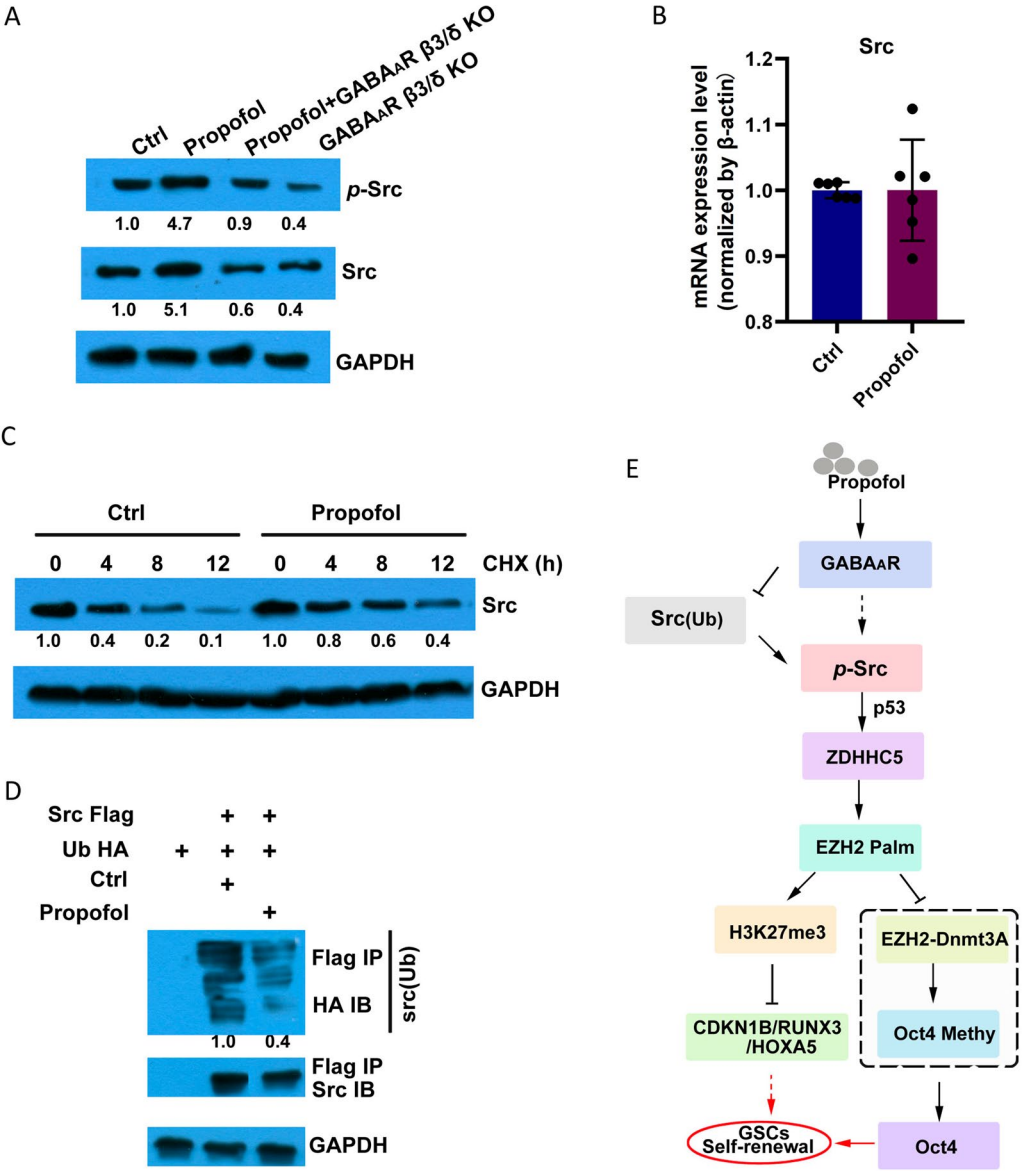
Propofol increases Src expression via GABA_A_R. **A** Western blotting for *p*-Src and Src in wild-type or GABA_A_R *β*3/*δ* knockout (KO) U118-derived GSCs treated with propofol (*n* = 5 in each group). GAPDH was used as a loading control. **B** The mRNA levels of Src in U118-derived GSCs treated with or without propofol were analyzed by RT-PCR (*n* = 5 in each group). *β*-actin was used as the loading control. **C** CHX chase assay indicates that the half-life of Src protein was markedly prolonged, by western blot analysis, with the treatment with muscimol or propofol compared to control conditions. GAPDH was used as the loading control. **D** Western blot analysis of Src ubiquitylation in U118-derived GSCs following the overexpression of Src-Flag or control vector and treatment with propofol or control condition. Src was immunoprecipitated with anti-Flag and immunoblotted with an anti-HA antibody. **E** Schematic representation demonstrating that propofol can enhance the stem-like properties of gliomas through the interaction of the GABA_A_R-Src-ZDHCH5-EZH2 signaling axis. Ctrl, control; *p*-Src, phosphorylated Src; and CHX, cycloheximide

Propofol treatment upregulated the levels of Src and phosphorylated Src (*p*-Src) in GSCs (Fig. 5A). Knockout of GABA_A_R subunits *β*3 and *δ* decreased Src protein levels and blocked the function of propofol in upregulating the expression of Src and *p*-Src (Fig. 5A). However, propofol did not significantly affect Src mRNA expression (Fig. 5B). Furthermore, the experimental half-life of Src protein was markedly prolonged (estimated from 4 to 8 h in this study) with propofol treatment in the CHX chase assay even at 12 h after propofol treatment (Fig. 5C). Notably, propofol inhibited the ubiquitylation of Src (Fig. 5D). These data suggest that propofol inhibits Src ubiquitination by acting on GABA_A_R, leading to Src accumulation, which promotes the growth of GSCs.

In summary, propofol can enhance the stem-like properties of gliomas through the interaction of GABA_A_R, Src ZDHCH5, and EZH2. Propofol activation of GABA_A_R increased Src expression, thereby enhancing palmitoylation of ZDHHC5-mediated EZH2 and Oct4 expression (Fig. 5E).

## Discussion

Propofol is a commonly used anesthetic. However, its effects on glioma growth and recurrence remain largely unknown. Here, these results suggest a potential pathway by which propofol activates GABA_A_R, leading to the upregulation of Src and subsequent upregulation of EZH2 palmitoylation mediated by ZDHHC5 and the enhancement of stem-like properties of gliomas that may promote tumor growth.

This study found that treatment with a standard dose of propofol promoted glioma growth in nude mice compared to control or low-dose propofol, because propofol treatment increased the stem-like properties of the tumor tissue. We compared the effects of low- and standard-dose propofol on tumor growth with different doses of propofol injected through the tail vein. However, this setting could not allow us to determine the roles of propofol anesthesia versus non-anesthesia condition because we could not use the pump to administer the same volume (600 µL) of intralipid (the vehicle of propofol) via tail vein without general anesthesia over 1 h. The injection of patient-derived cells (GBM0378) without intralipid to mice would cause confounding influence because no vehicle of propofol was administered to the mice. We, therefore, used low-dose propofol as the control condition in the experiment, which also represented the clinical condition for patients receiving standard-dose propofol for general anesthesia or low-dose propofol for sedation during regional anesthesia [34, 35].

To compare the effects of propofol anesthesia with those of non-anesthesia conditions on GSCs, we established another system in which GSCs were pre-treated with propofol dissolved in DMSO with DMSO alone for 6 h. We then injected these pre-treated tumor cells into mice and found that propofol-treated GSCs also led to larger tumor growth in nude mice than did vehicle-treated tumor cells. These results further suggest that propofol promotes the self-renewal and tumor-initiating ability of GSCs compared with the non-anesthetic status.

At present, the role of propofol in glioma is still controversial. Some research groups found that propofol could inhibit the proliferation and invasion of glioma cells by inhibiting the PI3K/Akt-ROCK1 signaling axis. However, we found that propofol has the potential to enhance tumor growth. The main reasons for the contradictions in the results are as follows: first, the difference in the concentration selection of propofol in the experiment. In animal trials, we have used standard injection dose and low dose to mimic clinical use. In vitro cell trials, we have used 4 µg/ml (the clinically relevant blood concentration of propofol). In the experiments of other research groups, most of the propofol dose of 5–10 ug/ml was adopted, which actually exceeded the actual clinical use or the propofol concentration in patients [36–38]. In fact, high concentrations of propofol also inhibited the self-renewal and tumorigenicity of glioma stem cells (Additional file 1: Fig. S5). Second, the differences in cell types used in the experiment. We mainly analyzed the regulation of propofol on the self-renewal of GSCs. However, other research groups mainly studied the biological regulation of propofol on glioma cells. Third, the complexity of biological functions. Another study reported that propofol (2–10 µg/mL for 6 h) promotes migration and invasion of oral squamous carcinoma cells [38], propofol (2–10 ìg/mL for 1–12 h) increases proliferation and migration of human breast tumor cells [39], and propofol (6 µg/mL) enhances migration of breast carcinoma cells [40]. This shows the complexity of propofol’s effects on tumors, which requires us to reveal its biological functions under characteristic scenarios.

After propofol treatment, the palmitoylation level of EZH2 in tumor stem cells increased, which may be related to the increased palmitoyltransferase ZDHHC5 expression. It has been reported that ZDHHC5 may be involved in the maintenance of dryness in glioma cells [25, 41]. In addition to the increased ability of GSCs to self-renew and the clone formation induced by propofol, we observed that propofol did not affect the level of unphosphorylated EZH2 but inhibited the phosphorylation of EZH2 S21 in GSCs. The effect of propofol on GSC growth was mainly due to changes in EZH2 activity rather than EZH2 expression. Notably, phosphorylation of EZH2 S21 was negatively correlated with EZH2 palmitoylation of ZDHHC5 at Cys 571 and Cys 576. Phosphorylation of EZH2 at S21 inhibits tri-methylation of H3K27, thereby inhibiting the expression of target genes of multicomb inhibitory complex 2, including p19, Bim, p57, e-cadherin, and RUNX3 [25, 42].

Mechanistically, propofol enhances stem cell-like properties of gliomas through GABAAR-mediated changes in Src expression. Upregulation of Src expression critically contributes to Src activation, which also could be detected in many tumors including colon, lung, breast, and endometrial tumors. Src is a non-receptor cytoplasmic kinase that mediates formation of focal adhesions and arrangement of actin filaments for cell extension, and Src activation contributes to epithelial-mesenchymal transformation, a key event in tumor stem cell development [43, 44]. In GBM, Src can physically interact with the transcription factor p53, which leads to the abnormal upregulation of ZDHHC5, whose promoter contains four potential Src/p53 binding sites. A recent report showed that GABA_A_R activation reduces the expression of cytosolic ubiquitin ligase TRIM21, inhibits Src ubiquitination, and leads to Src accumulation [10]. Unfortunately, no direct interaction between E3 ligase TRIM21 and Src in co-IP determination has been observed to date, suggesting that Src degradation may be mediated indirectly by TRIM21.

## Conclusions

This study suggests that propofol, a commonly used anesthetic, can promote the self-renewal and tumor initiation of GSCs in mice via the GABA_A_R-Src-ZDHHC5-EZH2 signaling axis. These findings may lead to more research into anesthesia and tumor growth in preclinical and clinical settings, ultimately leading to better outcomes in patients with surgically removed tumors.

## Supplementary Information

**Additional file 1**. Supplementary Figures.

**Additional file 2**. Supplementary Table.

## Data Availability

All data generated or analyzed during this study are included in this published article [and its Additional files 1 and 2].

## Abbreviations

ABE: Acyl-biotin exchange method
ChIP: Chromatin immunoprecipitation
Co-IP: Co-immunoprecipitation
EZH2: Enhancer of zeste 2 polycomb repressive complex 2 subunit
GABA_A_R: Gamma-aminobutyric acid A receptor
GSC: Glioma stem cell
HAM: Hydroxylamine
LC–MS-MS: Liquid chromatograph-mass spectrometer-mass spectrometry
Src: Src proto-oncogene, non-receptor tyrosine kinase
RT-PCR: Quantitative real-time polymerase chain reaction
ZDHHC5: Zinc finger Asp-His-His-Cys-type palmitoyltransferase 5
