# Supplementary material for: Propofol enhances stem-like properties of glioma via GABAAR‐dependent Src modulation of ZDHHC5-EZH2 palmitoylation mechanism
Source: Stem Cell Res Ther. 2022 Aug 4;13:398. doi: 10.1186/s13287-022-03087-5 (PMC9351178; doi:10.1186/s13287-022-03087-5)
Supplement: Supplementary file 1 — Additional file 1. Supplementary Figures. [file 13287_2022_3087_MOESM1_ESM.docx]

SUPPLEMENTARY INFORMATION

**Propofol enhances stem-like properties of glioma via GABA_A_R‐dependent Src modulation of ZDHHC5-EZH2 palmitoylation mechanism**

Xiaoqing Fan^1,*^, Meiting Gong^2^, Huihan Yu^2^, Haoran Yang^3^, Sheng Wang^1,*^, RuitingWang^1,*^

^1^Department of Anesthesiology, The First Affiliated Hospital of USTC, Division of Life Sciences and Medicine, University of Science and Technology of China (USTC), No. 17, Lujiang Road, Hefei, Anhui, 230001, China

^2^Department of Pathophysiology, School of Basic Medicine, Anhui Medical University, No. 81, Meishan Road, Hefei, Anhui, 230032, China

^3^Department of Molecular Pathology, Hefei Cancer Hospital, Chinese Academy of Sciences, No. 350, Shushan Hu Road, Hefei, Anhui, 230031, China

***Corresponding author**: Dr. Xiaoqing Fan (15055701159@163.com), Pro. Sheng Wang (iamsheng2020@ustc.edu.cn), and Pro. Ruiting Wang (ruitwang@163.com)

**Supplemental Figure 1**

**
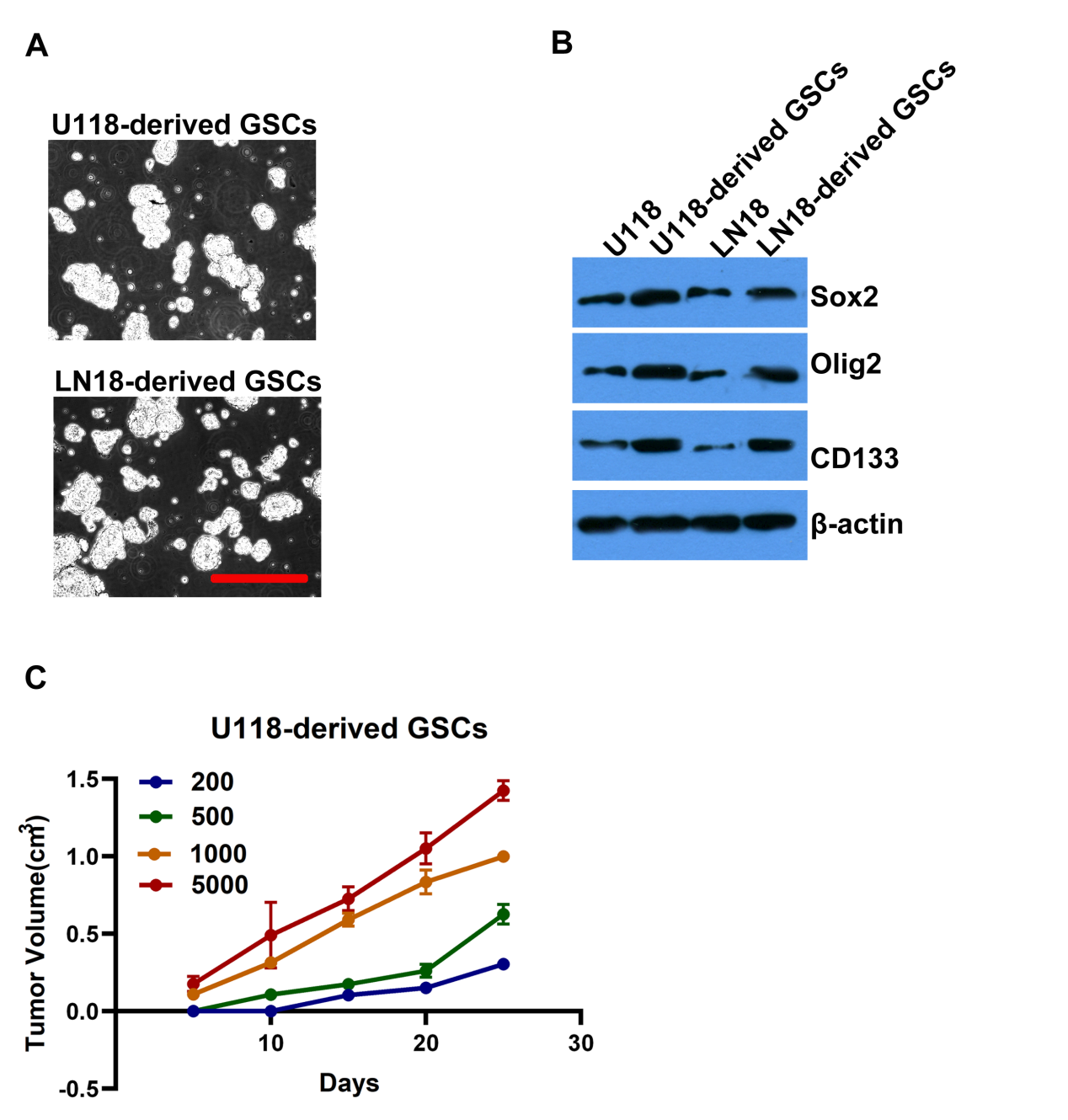
Supplementary Figure 1. Biological characteristics of GSCs derived from U118, and LN18 glioblastoma cells.** (A) The U118-derived and LN18-derived GSCs have the sphere-formation ability. Scale bars, 200 μm. (B) The expression level of stemness markers (Sox2, Olig2, and CD133) were upregulated in GSCs, compared to glioblastoma cells. (C) GSCs have the tumorigenic potential. When the number of cells inoculated was more than 200, the U118-derived GSCs could obviously form tumors.

**Supplemental Figure 2**


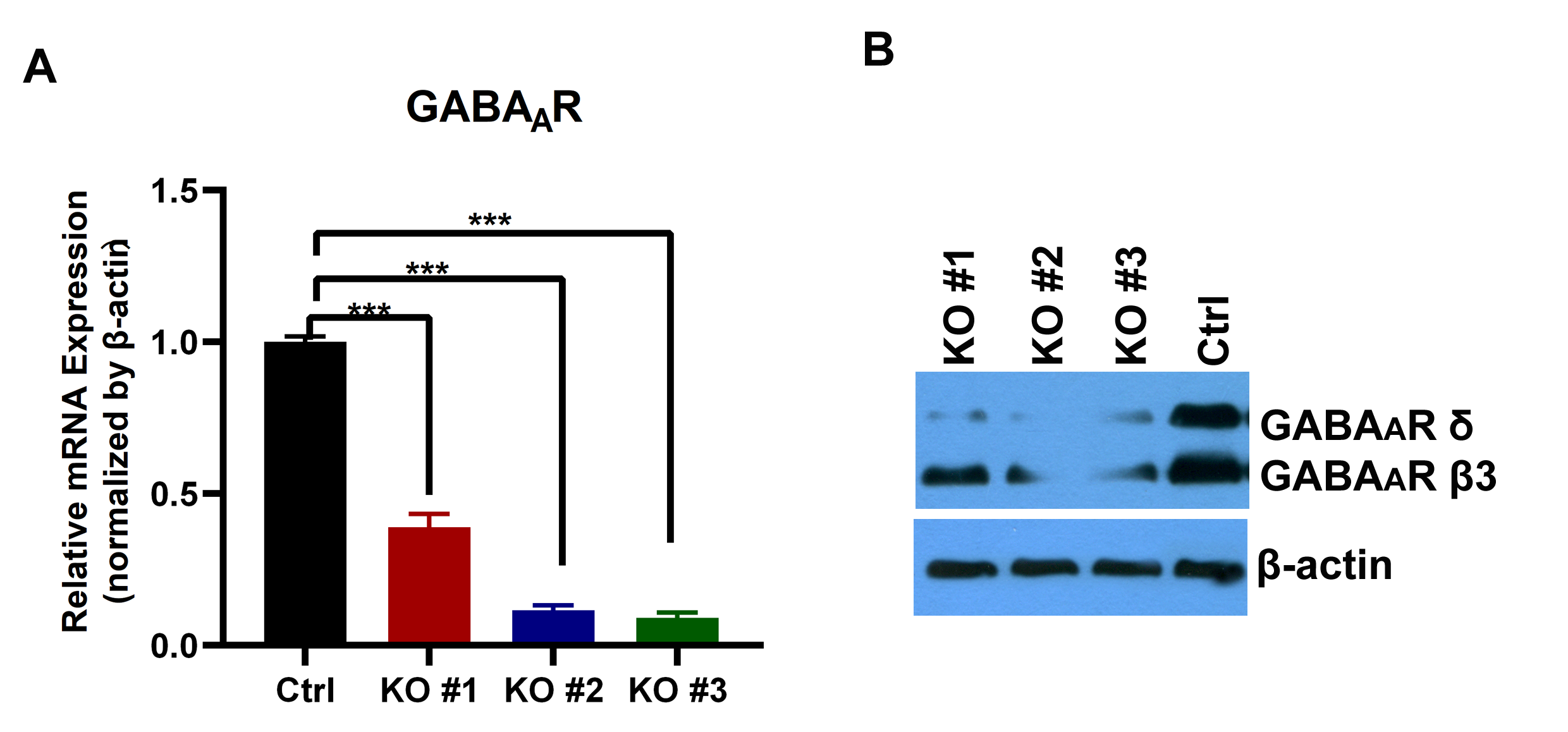
**Supplementary Figure 2. Identification of GABA_A_R knockout effect.** The mRNA (A) and protein (B) levels of GABA_A_R in GABA_A_R knockout U118-derived GSCs colony (#1, #2 and #3) were analyzed by RT-PCR, and Western blot, respectively. KO, knockout.

**Supplemental** **Figure 3**

**Supplementary Figure**
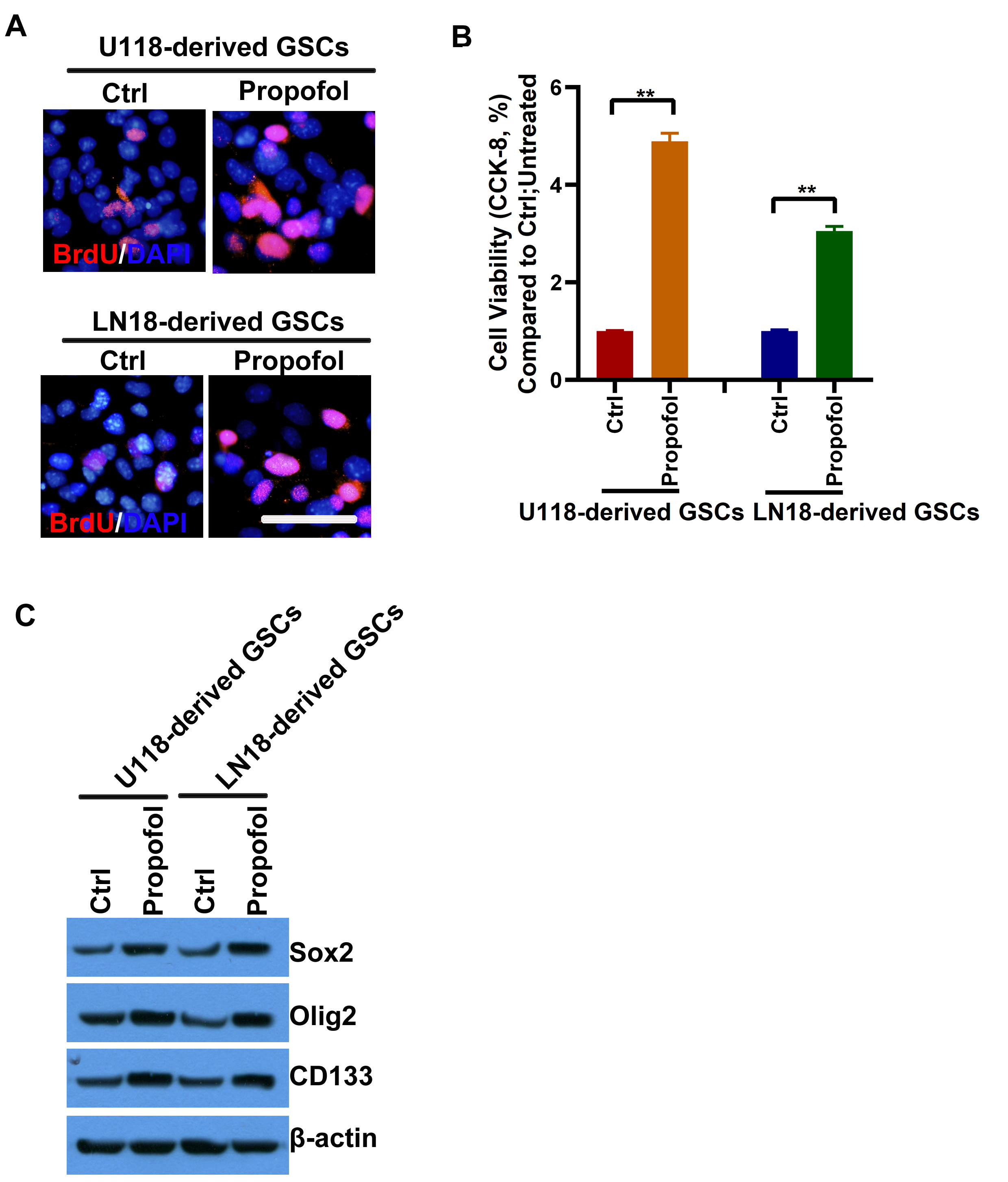
**3. The self-renewal was enhanced in U118-derived and LN18-derived GSCs after propofol treatment.** (A) The proliferation ability of U118-derived and LN18-derived GSCs treated with propofol was analyzed by BrdU assay. Scale bars, 100 μm. (B) The metabolic activity in U118-derived and LN18-derived GSCs treated with propofol was analyzed by CCK-8 assay. (C) The expression level of stemness markers (Sox2, Olig2, and CD133) in U118-derived and LN18-derived GSCs treated with propofol was analyzed by Western blot.

**Supplemental** **Figure** **4**

gtgagggatgcgggcgcccatcccacgtgtgacagtgggaaactaatggaattcagggagttcacttcccggctcgcactccgggggcgggagggtgctgccaccgcctcaggtcgcgcgctccttaggcttccctctcacggttagcgctacggctccaaggctggcgcctgctcttcttcatctccctccccggcccttggcaggagcctcgccctacattggctcttcaagtccgggcgtccccttcatgtgggagacacagcccagaccattccatcgccctggattctgcccggaccagcgacctcgccggactgaagatttctccagcaagattgttttaaataaagtttttgccatcaaaaaaaaaaaaaaaaaaaaaaaaggagatgggaaggagaagagggaggcctagtcgccaaatggttgg**ccaat**ccgccgcgaaggggctgaagcaggaagcggcgatagtcgtcggtaattggctgcgctggaagcctgtctggactgtaccacctcagtaggagaggggttgctgaattcgagaaggtagctgcttgcttttgagtacttctagtacacacacataattcttttcttatttccgggggtttttgcttggattcagtgggaatacaacaggacgcaggagaagggaagactcgcctggccatttcctcattcctgactcctgtgcattagg**ccaat**ccttgtctcttagttgtccccgcaaccccacggccttacgtgcgcgctctcgtcttctcgcgtgcgtacgtcgttgtgttgctgcag**ccaat**caggccgcgaggtcgccgcggtcgccagggttcgcgcgcacgccccagatcggagtcggggtgggggctatgggtgaatgggagagtgagcggggttgggcgcggcagcgcgagccgcatgaatgagacaaacgtgcgccgcgcgagacgtcgcgcgagcccggactagggag**ccaat**gggaacgctggaagggttc

**Supplemental** **Figure** **4.** **There** **are** **four** **potential** **binding** **sites** **(red)** **for** **Src/p53** **in** **the** **1kb** **upstream** **region** **of** **the** ***ZDHHC5*** **promoter.**

**Supplemental** **Figure** **5**

**
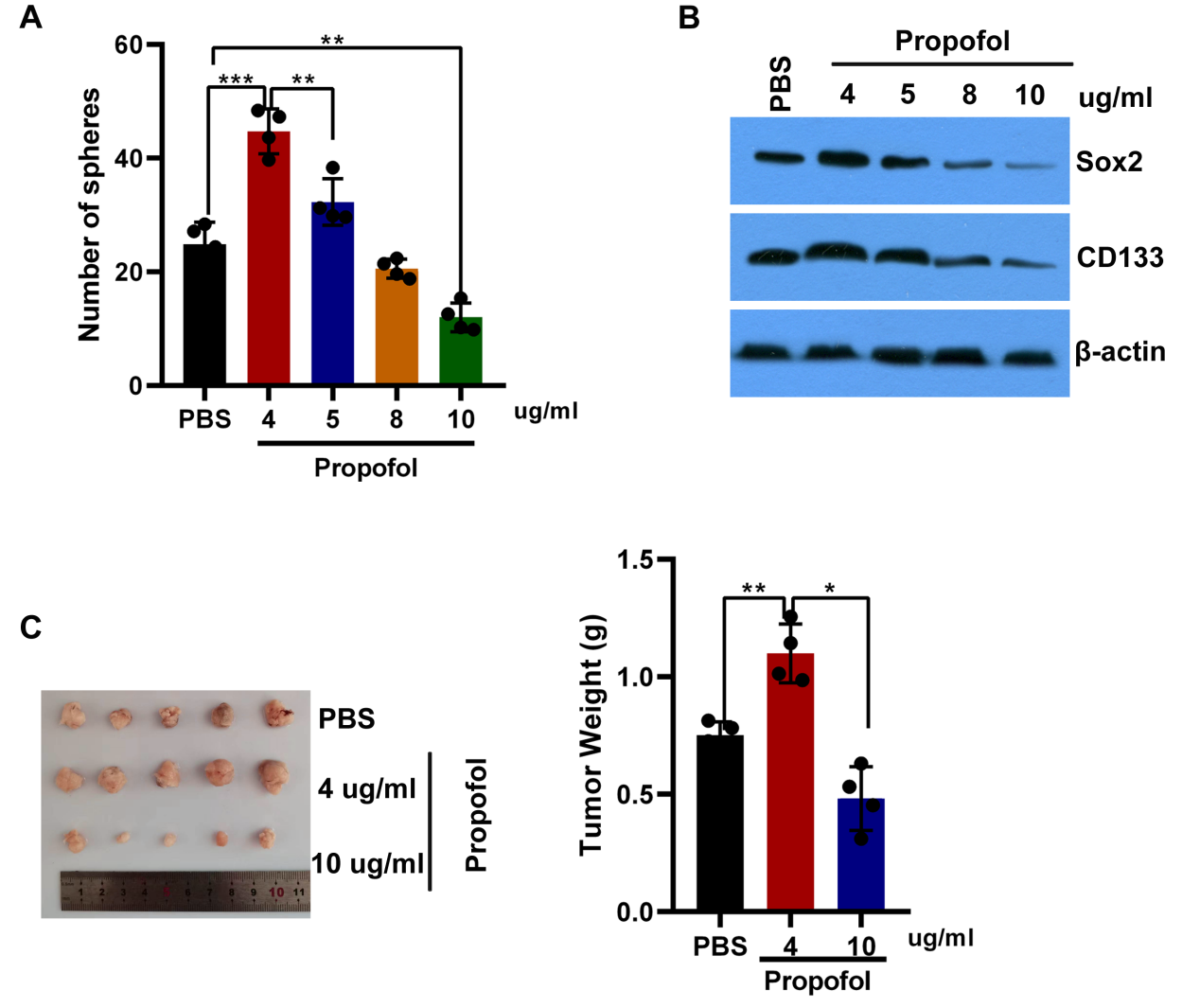
**

**Supplemental Figure 5. High-dose propofol inhibited the self-renewal and tumorigenicity of glioma stem cells.** (A, and B) Detection of glioma spheres formed for U118-dervied treated with or without different concentrations of propofol and cultured for a second passage. Scale bars, 100 μm. Quantification of glioma spheres formation capacity (*n*=5 in each group). (C) Tumor weight of U118-dervied GSCs in BALB/c mice intraperitoneally administered with different concentrations of propofol on days 6, 12, and 18 post implantation. Tumor weights were measured after 20 days (*n* = 5 mice/group).
